# Integrating plasticity into precision psychiatry

**DOI:** 10.1192/j.eurpsy.2025.19

**Published:** 2025-02-12

**Authors:** Igor Branchi

**Affiliations:** Center for Behavioral Sciences and Mental Health, Istituto Superiore di Sanità, Rome, Italy

**Keywords:** environmental interventions, lifestyle, network theory, plasticity measurement, transition

## Abstract

Understanding transitions from psychopathology to well-being is crucial for promoting recovery. Plasticity – the ability to modify brain functioning and mental states – is increasingly recognized as essential because it enables the reorganization of neural and mental processes underlying such transitions. Recently, a network-based approach that operationalizes plasticity, and the ability to transition to well-being, as the inverse of the connectivity strength within the symptom network has been proven effective in predicting both the likelihood and timing of recovery from major depressive disorder. This innovative method to measure plasticity is opening new avenues for timely diagnosis, patient stratification, and targeted, individualized treatment of mental illness. Overall, integrating the assessment of plasticity levels into precision psychiatry holds significant potential for developing novel and effective personalized therapeutic strategies in psychiatry.

## Plasticity as a critical factor to achieve mental well-being

In psychiatry and neuroscience, plasticity is defined as the ability to modify brain functioning and mental states [1]. It arises from processes occurring across multiple scales, from the molecular to the behavioral one. Plasticity is increasingly acknowledged as a crucial process in the recovery from psychiatric disorders because it underlies the reorganization of neural and mental processes during transitions from psychopathology to well-being [2].

## Plasticity is not inherently beneficial: the relevance of context

**Figure 1.**
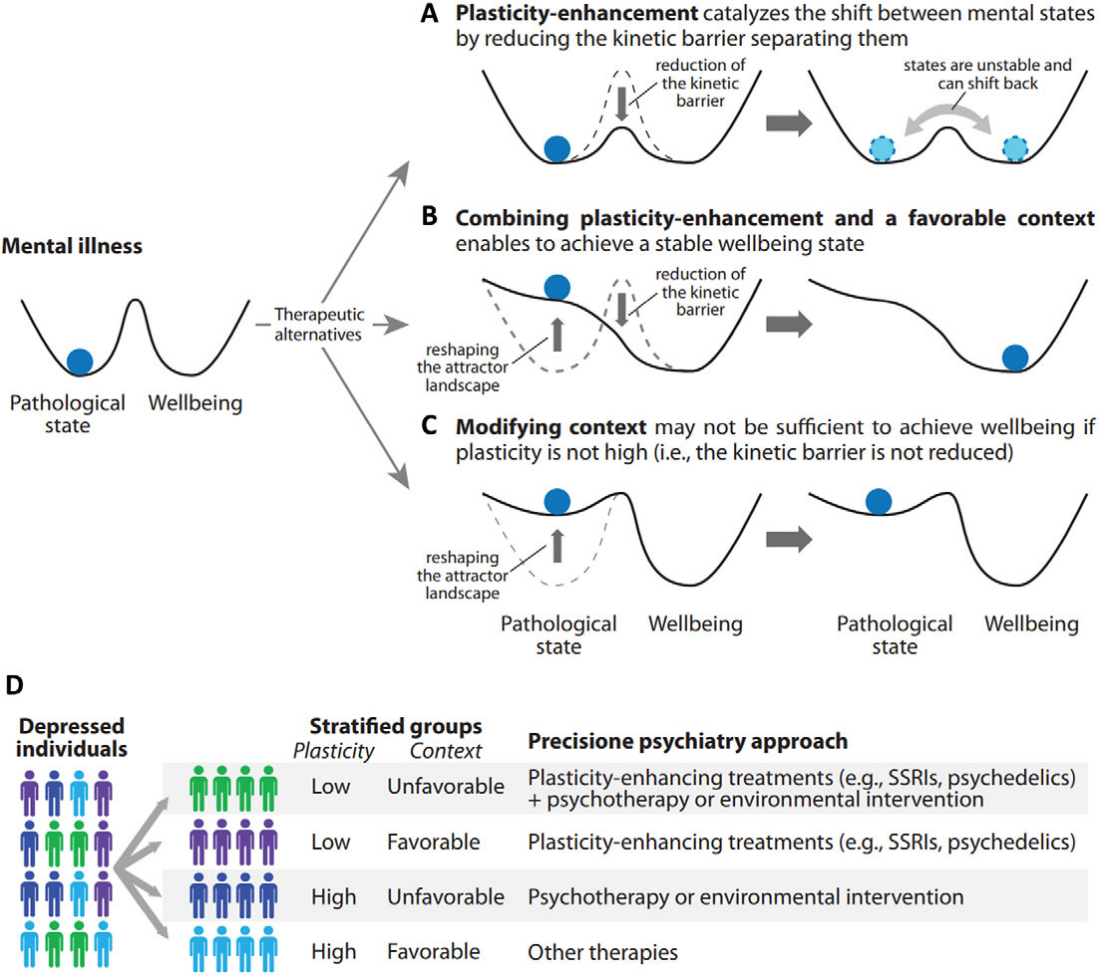
Overview of the role of plasticity and its interplay with context in the transition from psychopathology to well-being. A landscape with valleys representing different mental states, such as psychopathology and well-being. The hills between them represent barriers that hinder the transition from one state to another. The therapeutic goal is to help the system (i.e., an individual) transition from a pathological state to well-being, which can be imagined as a ball rolling from one valley to another. (**A**) Enhancing plasticity enables the transition but does not promote the stability of a specific mental state. (**B**) Combination of high plasticity and a favorable context is the most effective therapeutic strategy as it enables the transition and promotes sustained well-being. (**C**) The action exerted by the context can stabilize well-being but it may be not sufficient to achieve it. Adapted from Branchi I, Giuliani A. Shaping therapeutic trajectories in mental health: Instructive versus permissive causality. European neuropsychopharmacol 2021; 43:1–9. (**D**) Personalized therapeutic strategies within a precision psychiatry approach. By tailoring interventions to both a patient’s plasticity level and quality of contextual enables effective therapies aimed at maximizing recovery potential.

It is noteworthy that the above definition implies that plasticity is neither inherently beneficial nor detrimental as an enhancement of plasticity increases the likelihood of mental state transitions without determining the direction in which these transitions occur (Figure 1A). The direction is determined by moderators including contextual factors, such as living conditions or subjective appraisal of quality of life [1]. Accordingly, evidence increasingly demonstrates that treatments able to enhance plasticity produce effects that can be highly context-dependent, amplifying the influence of contextual factors in shaping mental health and behavioral outcomes. Consequently, high plasticity has a greater therapeutic impact when combined with supportive living conditions or psychotherapeutic approaches [1–3] (Figure 1B). Indeed, the efficacy of treatments such as selective serotonin reuptake inhibitors (SSRIs), psychedelics and ketamine – that reportedly enhance plasticity – is dependent, at least partially, on pairing them with favorable environmental conditions [2, 4, 5]. Accordingly, the combination of antidepressants with psychotherapy is more effective than the drugs alone [6].

## Plasticity levels as a key determinant of interindividual variability in the efficacy of environmental, lifestyle, and psychotherapeutic interventions

Therapeutic strategies based on lifestyle, environmental, or psychotherapeutic interventions are increasingly recognized as essential for promoting mental health. However, not everyone benefits equally from these approaches. For many individuals, targeting the living conditions [7] or subjective appraisal [6] may not be sufficient. This disparity can be attributed, among other factors, to differences in plasticity. Individuals with high plasticity can swiftly modify their mental state in response to these interventions (Figure 1B), while those with low plasticity cannot [2] (Figure 1C). Therefore, the outcome of plasticity on mental health depends on the context and vice versa, making their interplay highly relevant for developing personalized therapeutic strategies in psychiatry. Specifically, fostering high plasticity with supportive contextual conditions is essential for promoting recovery and well-being. In clinical settings, it is thus necessary to assess both an individual’s quality of context and plasticity levels. While psychometric tools to evaluate the quality of context, such as quality of life questionnaires, have long been available, methods to assess and operationalize plasticity remain limited. Only recently innovative strategies have started to emerge.

## Operationalizing plasticity: measuring susceptibility to change mental state

Plasticity can be assessed through various advanced methods. Neuroimaging techniques, such as functional magnetic resonance imaging (fMRI), track changes in brain activity over time. Electrophysiological techniques like electro-encephalograms (EEG) measure real-time neural responses, helping assess plasticity at the cortical level [8]. These tools have been instrumental in demonstrating how the brain can undergo structural and functional changes in response to various experiences, and in effectively assessing neuronal coherence and connectivity [9, 10]. Yet, these techniques are limited in depicting real-time plasticity changes because of the suboptimal space resolution of EEG and insufficient temporal resolution of fMRI. An additional limitation, particularly for fMRI, is its relatively high costs and practical challenges in implementation. Moreover, for effective clinical application, these approaches should measure plasticity encompassing an individual’s overall ability to modify their mental state to transition from psychopathology to well-being. Therefore, novel strategies to operationalize plasticity are still warranted.

The seminal works by Denny Borsboom on the network theory of psychopathology [11] have been among the most innovative scientific ideas in the mental health field in recent years. By conceptualizing mental disorders as networks, the onset, progression, and recovery of psychopathology can be explored by exploiting the general properties of networks and graphs [11]. Building on this theoretical framework, the *network theory of plasticity* has been recently introduced [2]. This theory proposes the connectivity strength among the elements of a system as a measure of system plasticity and thus of its ability to change its outcome. In a highly connected network, each element is limited in its ability to change as its modifications are constrained by the necessity of simultaneously modifying all the other connected elements. Conversely, in a weakly connected network, each element can be modified with limited or no constraints. Plasticity has thus been operationalized as the inverse of connectivity strength. When conceptualizing an individual as a network of interconnected symptoms, the individual’s plasticity – and thus their ability to transition from psychopathology to well-being – is predicted to be inversely related to the connectivity strength within the symptom network. For instance, in the case of studies on depression, connectivity has been measured as the sum of correlations, reflecting the overall degree – whether positive or negative – of co-occurrence among the nine standard symptoms of major depressive disorder as defined by the DSM-5. The validity of this operationalization has been recently demonstrated through an analysis of two independent datasets, the STAR*D-Sequenced treatment alternatives to relieve depression and the CO-MED-Combining medications to enhance depression outcomes [12, 13]. Findings revealed that baseline connectivity strength among symptoms is significantly weaker in responders than in nonresponders (e.g., those not experiencing significant improvement after an adequate course of treatment). This difference reflects the higher plasticity and greater capacity for change in depression scores observed in responders. Moreover, baseline connectivity strength inversely correlates with subsequent improvement over 4 weeks: the weaker the connectivity – and thus the higher the plasticity – the larger the improvement in depression score. As high plasticity promotes changes in mental states according to contextual factors, baseline connectivity strength correlates with the susceptibility to change depression score according to the quality of context both in patients showing an improvement or a deterioration of the symptomatology. Finally, the operationalization of plasticity exhibited high sensitivity, effectively differentiating individuals based on the timing of their recovery trajectory [13]. Further investigations are warranted to consolidate the reliability of these findings and identify the limitations of the approach. Overall, the network-based operationalization of plasticity represents a novel mathematical tool for understanding and predicting resilience, vulnerability, and capability to recover. In addition, it holds promise to improve approaches to prevent and treat depression. As the measure of plasticity pertains to basic features of complex systems, it is likely generalizable at multiple levels of analysis, from the symptomatology to the neural features, and across diseases.

## A precision psychiatry approach: stratifying patients by plasticity and context

By assessing individual plasticity levels through its operationalization and evaluating quality of context using existing questionnaires, patients can be stratified according to both plasticity and context. This stratification can be leveraged to design targeted therapeutic strategies within a precision psychiatry approach. For instance, patients with high plasticity are expected to possess the potential for transitioning to well-being. However, if they experience unfavorable contextual factors, they need to undergo therapies to improve their quality of life to harness their ability for a beneficial outcome, such as lifestyle interventions or psychotherapy. By contrast, patients with low plasticity are expected to show no or slow transition to well-being even if exposed to supportive conditions. In this case, the transition toward well-being might be promoted by treatment with SSRI and psychedelics [1, 4] or, more in general, by approaches able to enhance plasticity [14] (see Figure 1D for further details).

Further potential applications of plasticity assessment in clinical settings stem also from viewing the time required to shift from one state to another, such as from psychopathology to well-being, related to plasticity levels: the higher the plasticity, the faster the transition. Indeed, a recent study based on the network-based operationalization of plasticity has shown that plasticity levels at baseline, measured as connectivity strength, predict the time to both clinical response and remission [13]. This approach promises to identify disease trajectories at enrollment, leading to tailored approaches.

## Harnessing plasticity to promote mental health

In conclusion, the conceptual shift from viewing plasticity as an instructive factor driving toward recovery, to a permissive factor determining the influence of the contextual factors on mental health [2], provides a novel theoretical framework that holds promise for advancing psychiatry and the understanding of mental illness. In addition, emerging strategies, such as the network-based operationalization of plasticity, that provide a quantifiable measure of plasticity – and thus of the ability to change mental state – pave the way for personalized preventive and therapeutic approaches within precision psychiatry.

Finally, the perspective proposed here not only underscores the importance of integrating plasticity into clinical practice but also emphasizes the relevance of contextual factors, including the individual subjective appraisal of their quality of life, when assessing the efficacy of psychopharmacological interventions. Overlooking the drug by context interplay may partly explain the high variability in the efficacy of pharmacological treatments, especially those affecting plasticity levels such as classic and novel antidepressants. This oversight could represent one of the causes that has contributed to low trial sensitivity leading to a progressive decline in the investments for the development of pharmacological approaches in psychiatry and brain health. By incorporating plasticity and context as key elements in the drug development process, there is potential to reinvigorate research and attract new investment to ultimately advance treatment options for psychiatric patients.
